# Supplemental Learning in Respiratory Physiology for Healthcare Professionals Towards Successful Treatment of COVID-19

**DOI:** 10.3389/fphys.2021.624528

**Published:** 2021-04-20

**Authors:** Helen Wallace, Robert Angus

**Affiliations:** ^1^Department of Women and Children’s Health, Institute of Life Course and Medical Sciences, University of Liverpool, Liverpool, United Kingdom; ^2^School of Medicine, Institute of Life Course and Medical Sciences, University of Liverpool, Liverpool, United Kingdom

**Keywords:** education, respiratory, physiology, clinical, COVID-19, pathophysiology, treatment

## Abstract

The immunological and pathophysiological response to COVID-19 can cause severe respiratory impairment affecting gas exchange and lung mechanics. Such was the scale of the respiratory support needed during the first wave of the pandemic, that recruitment of non-respiratory clinical staff was essential to help deal with the growing number of cases. It quickly became apparent that it was vital to rapidly equip these healthcare professionals with appropriate physiological knowledge and practical skills if therapies were to be applied effectively. Furthermore, the unravelling of unusual clinical features of COVID-19, further highlighted a need for knowledge of long-established principles of respiratory physiology. An online digital educational resource, or “respiratory learning tool kit” was developed with interactive material including visualisations, animations, and pathophysiological examples to facilitate understanding. The learning outcomes were centred on physiological principles, essential for understanding the pathophysiology relating to COVID-19, and management and treatment. Topics included principles of gas exchange, gas transport, homeostasis and central control of respiration. These basic physiological principles were linked to pathophysiology and clinical skills around oxygen administration and non-invasive supports such as Continuous Positive Airway Pressure (CPAP). From the degree of engagement and evaluation comments, it was clear that the resource successfully achieved its aim—to increase physiological knowledge and its practical understanding, enabling healthcare professionals to practice with confidence in such an uncertain environment.

## Introduction

On the 11th March 2020, the World Health Organisation declared coronavirus disease 19 (COVID-19) a pandemic, meaning the illness had spread worldwide. Coronavirus disease 2019 is a viral disease caused by a coronavirus named severe acute respiratory syndrome coronavirus 2 (SARS-CoV-2) (Chan et al., 2020). A mammoth effort has been made to characterise COVID-19, and while this knowledge is evolving weekly, less than a year in, the transmission, pathogenesis, clinical course and even longer term consequences are already described (Frazer, 2020; Muge et al., 2020).

In COVID-19 infection, the immunological response with its pathophysiological consequences can cause severe respiratory compromise affecting gas exchange and lung mechanics. This has resulted in a large number of people needing oxygen therapy or some degree of respiratory support during the pandemic, presenting major challenges to expert respiratory and critical care teams and acute clinicians. The scale of the pandemic was such that nurses, doctors and paramedical staff from outside areas of respiratory expertise moved to supplement these teams. It followed that there was an urgent need to understand the best modalities of support for people, and to solve issues of minimising aerosolisation and the potential for cross infection.

Training was an urgent need; in addition to practical clinical skills around oxygen administration and non-invasive supports such as Continuous Positive Airway Pressure (CPAP), an understanding of lung physiology and the optimisation of function with intervention was critical. While some clinicians such as theatre and anaesthetic teams had appropriate skills and knowledge, many of the clinicians assisting had nothing more than their pre-qualification education to fall back on. These clinicians needed the appropriate physiological knowledge as well as practical skills if therapies were to be applied effectively and patients properly monitored. In this article, we report on a “respiratory learning tool kit” that was developed to support healthcare professionals who found themselves in the unfamiliar territory of respiratory critical care. We outline key physiological principles and relate them to the pathophysiology that occurs as a result of COVID-19 infection. We then go on to demonstrate how this knowledge feeds into decisions on management and treatment of COVID-19 patients and how the toolkit provided the essential knowledge required.

### Key Physiological Principles Relating to Pathophysiology of COVID-19

One of the manifestations of COVID-19 disease consists of pulmonary injury and entry of fluid into alveolar spaces. The resulting effect on the respiratory system can only be completely understood by re-visiting first principles on gas exchange—the process of gas exchange including relevant gas laws and diffusion, and how the respiratory system is designed to maximise this process under normal conditions (see Supplementary Material). Understanding the concept of gas diffusion gradients and the oxygen (O_2_) alveolar-arterial (A-a) gradient is vital when faced with problem-solving clinical scenarios and when decision making on choosing the appropriate methods to improve lung mechanics and gas exchange. Of course, when considering the above, carbon dioxide (CO_2_) is also an integral part of gas exchange, and the regulation of CO_2_ levels and blood pH is vital to homeostasis and normal physiology. Moving on from gas exchange itself to O_2_ transport provides understanding on how O_2_ is delivered to the tissues. Here, the relationship between O_2_ and haemoglobin and knowledge of the O_2_ dissociation curve is key to understanding the dynamics of O_2_ availability and the thresholds that maintain normal physiological processes.

Work of breathing and compliance are important determinants of a patient’s respiratory course and symptomatology. Underlying physiological principles include the mechanics of breathing in relation to Boyle’s law; the airflow, resistance and pressure gradients across the lung; and lung volumes and capacities. Integral to the understanding of lung mechanics is the central control of breathing, and how ventilation is modulated under different conditions including feedback loops from central and peripheral chemoreceptors. Local regulation of ventilation and perfusion and ventilation-perfusion rate is another key determinant of O_2_ and CO_2_ levels in the blood.

In addition to the pathophysiology, basic physiological principles must be applied in order to understand how different therapies can be used to treat respiratory failure. Patients with COVID-19 mainly present with type 1 respiratory failure with low O_2_ levels and normal or low CO_2_. The World Health Organisation (WHO) interim guidance indicates that 15% of patients require oxygen therapy, and 5% need ITU support with modalities such as continuous positive airways pressure (CPAP) or invasive ventilation (WHO, 2020). When arterial O_2_ is low, the O_2_ gradient becomes important to determine the source of the hypoxemia and O_2_ therapy would be the most effective treatment. In instances where standard O_2_ therapy is insufficient to prevent respiratory distress, other more intensive treatments would need to be considered. Another interesting observation in COVID-19 patients, is that the proning technique can be successfully used to improve lung mechanics and gas exchange. In this technique, functional alveoli are recruited more effectively in ventilated patients.

What also became clear in certain patients with COVID-19 was that clinical picture and apparent recovery often did not follow the type of patterns that clinicians routinely see with acute respiratory infection. For example, with basic bed side observations such as respiratory rate or pulse, patients often appeared well with no signs of respiratory distress; and yet when measured had remarkably low O_2_ saturations. Indeed, in the popular press it was reported that patients with “remarkably low O_2_ saturation, seemingly incompatible with life and clearly at risk, were using their mobile phones” (Levitan, 2020). This phenomenon has been reported across the globe in patients with COVID-19 and has been referred to as silent hypoxemia (Chandra et al., 2020). Unlike most other acute respiratory diseases, oxygen levels can fall dangerously low, without initially causing respiratory distress. By the time symptoms manifest, patients will have become in need of urgent intervention. Hypoxemia, is pre-dominantly due to the compromise of alveolar spaces or possibly micro-emboli associated with COVID-19 (Muge et al., 2020), without an apparent early change in lung mechanics. Other pathophysiological mechanisms to explain the lack of dyspnoea, include the reduced response of the respiratory centres in the brain to low O_2_, particularly in elderly patients (Kronenberg and Drage, 1973; Peterson et al., 1981) and also in the presence of comorbidities like diabetes where responses are blunted (O’Donnell et al., 1988; Nishimura et al., 1989; Weisbrod et al., 2005). Again, referring back to key physiological principles, the sensitivity of respiratory centres to minute changes in CO_2_, even small increases in PaCO_2_ will result in large increases in ventilation. Carbon dioxide levels are often normal or low in patients with COVID-19, so the body will not be alerted that there is a problem. Altered central and peripheral chemoreceptor response could be postulated, with the SARS-CoV2 ACE2 receptor cells being found centrally (Xia and Lazartigues, 2008) and in the carotid body (Fung, 2015). Further potential mechanisms include the disproportionate chemoreceptor stimulation to oxygen saturation due to temperature-induced shifts to the right of the O_2_ dissociation curve; high temperature or fever is a pre-dominant feature of COVID-19. Finally, viral effects on blood vessel constriction through ACE2 receptor binding may contribute to hypoxemia by causing dysregulation of ventilation-perfusion coupling. Recognition of “silent” hypoxemia has emphasised the importance of physiological measurement, together with monitoring, to inform decisions on treatment and management. Understanding the underlying physiological mechanisms and how they are modified will serve to enhance patient care (Tobin et al., 2020).

Emerging evidence indicates that patients may experience respiratory symptoms months after contracting COVID-19 (Frazer, 2020). Predictions can also be made from the 2003 outbreak of severe acute respiratory syndrome (SARS). However many patients recovering from COVID-19 are suffering from pre-existing disease so they may demonstrate more severe long-term effects. Knowledge of lung physiology will be important in helping patients understand some post COVID features, where in some, the lung injury leads to fibrosis, altered compliance and impairment of gas exchange.

### Respiratory Learning Tool Kit

We were presented with the challenge of facilitating the acquisition of effective physiological knowledge and understanding, which could not be achieved by simply referring to standard textbooks or the plethora of often-unreliable information on the internet. We achieved this by developing a digital educational resource, that includes peer reviewed explanations of key physiological principles using visualisation and animations to facilitate understanding (Hwang et al., 2012; see Supplementary Material). Evidence has indicated that after testing knowledge, online eLearning for undergraduates in healthcare professions was equivalent or possibly superior to traditional learning (George et al., 2014). The digital physiology educational material was initially developed by the authors, as a supplement to the traditional didactic teaching of challenging key concepts for physiology and medical students. The digital resource was based on different learning approaches with a common structure, including learning through acquisition, investigation and practice (Laurillard, 2012). These core physiological principles were then linked to pathophysiological scenarios; explaining how disease processes can alter lung function and lung mechanics leading to respiratory failure, by giving clinical examples. The resource is also interactive, testing knowledge and understanding of the user as one progresses through each tutorial. This knowledge can then be applied to key features of COVID-19 as mentioned above. This is key to understanding the clinical review, assessment, monitoring and management of respiratory patients, some of whom may be at risk of developing respiratory insufficiency. The physiological learning outcomes included in the tool kit are detailed below:

• Define and describe the principles of gas exchange i.e., diffusion and the gas laws, and the properties of the alveolar-circulatory interface
• Explain the concept of homeostasis with reference to the respiratory system
• Explain the role of lungs in maintaining acid/base balance and explain how the kidneys interact with the lungs in acid-base regulation
• Know normal values of arterial blood gases and be able to interpret their basic values
• Discuss the central control of ventilation and the role of the medulla and pons in its control
• Describe how other brain regions including cerebral cortex, cerebellum, limbic system and hypothalamus can modulate ventilation
• Describe and explain the importance of how sensory input modulates ventilation including feedback loops from peripheral chemoreceptors and central chemoreceptors

Given the context, physiological principles were taught alongside the clinical teaching on therapeutics; physiologists, together with physicians, nurses and physiotherapists shaped and delivered the material. In the context of a pandemic of a novel organism, continuing education of healthcare professionals is vital for adequate prevention and management of disease. For example, CPAP and other aerosol generating procedures can generate small particles that travel and persist in the atmosphere. In order to reduce the risk of viral spread to staff and patients, treatment is delivered via a well-fitted full facemask, with a separate exhalation valve. Cohorting patients positive for COVID-19 is also carried out, and staff follow PPE guidelines meticulously when treating patients.

### Engagement and Evaluation of the Respiratory Tool Kit

To achieve the largest impact, the respiratory learning toolkit was put online, and enabled and curated by the third party charitable provider of education, “Education for Health.” The toolkit went live in July as the first wave of the pandemic was settling. Five Hundred and seventy three health care professionals accessed the website and utilised the content in the first 8 weeks. More detailed data obtained from 161 users, showed that 24 users completed the toolkit, while the remaining 137 did not complete the course but were noted to spend significant periods of time on the toolkit. It was possible to complete the toolkit in 1 day and the average time spent on the learning resource was 6.35 h ± 1.76 [(SEM) n = 161]. Users accessed the toolkit an average of 2.69 times ± 0.2, ranging between 1 and 14 times. One hundred users accessed the toolkit on the same day, with the remaining 61 users revisiting on a different day ranging from within 2 days to 5 months after the first access date. Feedback was invited after completion of the toolkit and was presented as a 5 point Likert scale as shown in Table 1. Levels of evaluation included “strongly disagree” through to “strongly agree”. The questions included, “The online session increased my knowledge and understanding of the subject,” and “I will be able to apply what I learned in my work.” Results are presented from the 26 users who evaluated the course. As can be seen in Table 1, none of the respondents scored “disagree” or “strongly disagree.” Overall, the number of people who completed the tool kit was extremely encouraging and more than expected. Of note, the time of day the toolkit was accessed varied; with earliest access noted to be at 03:49, median time of 14:28 and latest access at 22:47. This illustrates the flexible accessibility of the online learning tool. The high level of engagement and preliminary feedback indicates that the toolkit was very well received. Questions inviting free textual comments are shown in Table 2. Individual comments were extremely positive showing appreciation for both the physiological and clinical aspects of the course.

**TABLE 1:** Responses to evaluation questions.

**Question**	**Neutral**	**Agree**	**Strongly agree**	**No answer**
The online session increased my knowledge and understanding of the subject	3	10	12	1
I will be able to apply what I learned in my work	3	9	10	4
Total	6	19	22	5

**TABLE 2:** Free text comments to evaluation question.

**Question**	**Response**
Additional comments, this includes something you think could be added to/changed about the online content	“I think everything was up to date considering knowledge was limited at time of development of this course” “I feel very strongly that all staff working with acute Covid 19 patients should complete this course and it should be free to all Trusts to facilitate this. The quality of information is excellent and will go some way to ensuring that there is parity of knowledge across the NHS” “Short quizzes at the end of each section to test knowledge/learning” “Enjoyable course—easily completed in 1 day
Please tell us something you learned that you plan to put into practice in your work	“Correct management of the NIV patient, when to step up and step down patients to manage them effectively” “Acute Use of NIV and CPAP safely” “Learned a lot about NIV and respiratory care in relation to COVID-19” “More detailed questions to ask when assessing patients with breathlessness. I will be able to incorporate this into my patient assessments” “I will take a more active role in the setting up of patients on NIV/CPAP, ensuring correct settings, circuits etc.” “Understanding difference between BIPAP/CPAP” “Being clearer on the physiology behind respiration” “Always provide evidence based best practiced care to patients” “Looking more closely at blood gases”

## Discussion

The main objective in the development of this learning resource was to provide health care professionals with an immediate and easy access to a respiratory learning tool kit. Such was the observed lack of knowledge in the current pandemic, that there is an urgent need for the learning material. It must be emphasised that due to the circumstances surrounding the production of the learning material, the above initiative was not primarily intended as a full blown research study as reflected in the limited data obtained. There was no formal assessment of learners at the end of the course and no formal credit for the learning; the low numbers that completed the evaluation may reflect that. This is also a common outcome in other reported technology enhanced learning programs for healthcare professionals (Nicoll et al., 2018). Studies often provide insufficient data to support transferability or direct future learning programs, and it is difficult to ascertain whether e-learning positively affects patient outcomes (Vaona et al., 2018). Nevertheless, the data available from this respiratory learning toolkit clearly demonstrate a high level of engagement, and indications from evaluations suggest that the toolkit enhanced the knowledge and facilitated the understanding of the users in respiratory physiology (Tables 1, 2). As demonstrated from the large numbers who accessed and engaged in the tool kit, it was clear that the main aim was successfully achieved. Interestingly, although only a small proportion of users completed the toolkit, the average time spent on the resource was significant. This indicates that the users utilised content that was beneficial to themselves. Of further note, are the data showing the pattern of engagement, wherein a significant number of users re-visited the toolkit over a 5 month period. This suggests that users benefitted from the long term accessibility of this online platform, re-visiting the toolkit to consolidate their knowledge and understanding. Future studies will include more rigorous analysis of the self-assessment element of the toolkit to generate evidence of understanding. Further encouragement on the completion of the evaluation survey will also provide more information on the opinions and experience of users. It would be useful to determine which elements of the toolkit were utilised more than others.

The global COVID-19 pandemic requires a response from all aspects of life sciences. As described here, providing a combination of practical and physiological understanding acquired from the learning toolkit gives clinicians a foundational knowledge to practice with confidence in such an uncertain environment. As an example, silent hypoxia is described in some articles as “baffling.” With reflection, that need not be the case if one considers what is known about physiological response changes with age, temperature and comorbidities, in particularly diabetes. Severe acute respiratory syndrome coronavirus 2 may also have a direct effect on nerve function such as chemoreceptors, given the ACE2 receptor distribution and the effect of the virus on the olfactory nerve. The effect of coronavirus on chemoreceptors would indeed be a valuable research question that could partly explain the delayed pulmonary manifestation in patients. That said, the pattern of COVID-19 was something that needed to be described, learned and applied as effective management are evolved and researched. It is a credit to life sciences and medicine the way this has happened and continues.

This collaboration of university staff, clinicians and the charity team allowed the provision of a digital resource showing a high degree of engagement; it was easily accessible on any device, anytime, anywhere. Going forward, effective assessment and evaluation tools must be included in online learning tools to demonstrate knowledge acquisition and the overall degree of learner satisfaction. Further studies are also needed to assess the effects of technology enhanced learning on patient outcomes. Learning resources applying core physiological principles and practical application in this context can be expanded to a wider audience including post-graduate students, medical students and translational physiologists. The proper understanding of known physiology and pathophysiological responses should enhance patient management, focus research questions and certainly remove a degree of the “bafflingly.”

## Data Availability Statement

The raw data supporting the conclusions of this article will be made available by the authors, without undue reservation.

## Author Contributions

HW completed the physiological aspects of the respiratory toolkit, including animations, slides, audio and learning outcomes, and contributed to the writing of the manuscript. RA oversaw all aspects of the clinical content of the respiratory toolkit including identifying learning outcomes, and contributed to writing the manuscript. Both authors contributed to the article and approved the submitted version.

## Conflict of Interest

The authors declare that the research was conducted in the absence of any commercial or financial relationships that could be construed as a potential conflict of interest.
